# Back to BAC: The Use of Infectious Clone Technologies for Viral Mutagenesis

**DOI:** 10.3390/v4020211

**Published:** 2012-02-03

**Authors:** Robyn N. Hall, Joanne Meers, Elizabeth Fowler, Timothy Mahony

**Affiliations:** 1 School of Veterinary Science, The University of Queensland, Gatton, QLD 4343, Australia; Email: r.hall4@uq.edu.au (R.N.H.); j.meers@uq.edu.au (J.M.); 2 Queensland Alliance for Agriculture and Food Innovation, The University of Queensland, St Lucia, QLD 4072, Australia; 3 Queensland Agricultural Biotechnology Facility, Ritchie Laboratories, St Lucia, QLD 4067, Australia; Email: elizabeth.fowler@deedi.qld.gov.au (E.F.)

**Keywords:** chromosomes, artificial, bacterial, recombination, genetic, mutagenesis, cloning, molecular methods, transposition, DNA viruses, infectious clone

## Abstract

Bacterial artificial chromosome (BAC) vectors were first developed to facilitate the propagation and manipulation of large DNA fragments in molecular biology studies for uses such as genome sequencing projects and genetic disease models. To facilitate these studies, methodologies have been developed to introduce specific mutations that can be directly applied to the mutagenesis of infectious clones (icBAC) using BAC technologies. This has resulted in rapid identification of gene function and expression at unprecedented rates. Here we review the major developments in BAC mutagenesis *in vitro*. This review summarises the technologies used to construct and introduce mutations into herpesvirus icBAC. It also explores developing technologies likely to provide the next leap in understanding these important viruses.

## 1. Introduction

Members of the viral family *Herpesviridae* are characterised by double-stranded DNA genomes ranging in length from 125 to 240 Kbp. The genetic stability and potential for incorporating large amounts of foreign DNA have made the herpesviruses a promising target for the development of gene therapy and vaccine vectors. For these purposes, replication-limited herpesvirus vectors have been developed through the deletion of an essential viral gene, which can subsequently be provided *in trans* to permit virus replication in a controlled or limited manner *in vivo* (for a review see [1]). While many early studies based on these techniques were successful, the scope of determining viral gene function was limited by two factors. Firstly, genes could only be manipulated using homologous recombination in virus-susceptible cells. To successfully use this approach, the cells had to be amenable to a method of introducing foreign genetic material, to permit the introduction of the DNA transgene-material encoding the required modification. Secondly, it was difficult to efficiently generate mutant viruses with altered or deleted genes that are essential for virus replication. These types of mutations required the co-delivery of functional copies of the deleted genes to permit virus replication. This was generally achieved by the generation of stably transformed cell lines that constitutively expressed the gene of interest or by using helper viruses. As a result of these limitations, the development of novel vectors and gene function studies could be achieved, however it was a time consuming process. These requirements severely limited the capacity to efficiently generate viruses with the desired mutations and deletions. In contrast, the generation of BAC mutants is a much quicker process. However mutations affecting essential viral genes still require the gene product to be provided in *trans* to generate infectious virions.

## 2. Infectious Clone Technologies

A key step in the development of herpesviruses as biological vectors, and herpes biology in general, was the development of infectious clone technologies. Initial efforts to improve the efficiency for herpesvirus genomic manipulation involved the use of *Escherichia coli* plasmid replicons known as cosmids. As these cosmids can only accommodate up to 45Kbp of foreign DNA, a series of vectors that contained overlapping segments of the herpesvirus genome were required to facilitate the generation of recombinant viruses [2]. Typically modifications were made to one fragment, followed by the rescue of infectious virus by introducing the complementing cosmids into cells to generate infectious virus.

Luckow *et al.* [3] were the first to demonstrate the cloning of the complete genome of a large double-stranded DNA virus as a bacterial artificial chromosome (BAC) vector. This was achieved by cloning a baculovirus genome to permit propagation and manipulation of the viral genome in *Escherichia coli* and subsequent rescue of infectious virus. Messerle *et al.* (1997) subsequently extended this technique to the herpesvirus family by cloning the complete genome of murine cytomegalovirus into a BAC [4]. This facilitated the recovery of infectious virus once reintroduced into susceptible cells. Since this initial study the genomes of at least 20 herpesviruses of human and veterinary importance and their derivatives have been used to create infectious clones using BAC technologies (icBAC) [5,6,7,8,9,10,11,12,13,14,15,16,17,18,19,20,21,22,23,24,25]. More recently, BAC technology has also been extended to other DNA viruses including poxviruses [26,27], and to RNA viruses including coronaviruses and flaviviruses, through the development of infectious cDNA clones [28,29].

## 3. Advantages of Bacterial Artificial Chromosomes

The cloning of large fragments of genomic DNA for the past three decades has underpinned advances made in genome sequencing, identification of the causative genetics of disease and the development of disease models. The three major vector types utilized in these studies are BACs, yeast artificial chromosomes (YAC) and mammalian artificial chromosomes. While each of these systems have their own advantages depending on the research question to be addressed, BACs are undoubtedly the system of choice for studying herpesviruses. BAC vectors are based on the *E. coli* fertility factor (F-factor) replicon which is maintained as a circular supercoiled extrachromosomal single copy plasmid in the bacterial host [2,30,31]. BACs can accept inserts up to 300 Kb in length, which is sufficient to allow the genomes of all known herpesviruses to be maintained as an icBAC. The principal advantage BACs have over the traditional YAC systems is stability of insert propagation over multiple generations. This is an essential property in the context of herpesvirus biology, as the genomes of many of these viruses contain a variety of repetitive sequence elements that could promote instability. However many studies have successfully propagated icBAC over multiple passages without detecting rearrangements [4,5,7,8,9,10,12,26,32,33]. Although YACs are capable of maintaining very large DNA inserts of up to 1Mb, they have numerous disadvantages, including instability, chimaerism and handling difficulties such as shearing of DNA [30,31]. 

The capacity to continually propagate a viral genome with high fidelity also provides the opportunity to explore other avenues of herpesvirus biology, compared to tradition cell-based methodologies. Each BAC clone represents a single replicative template that has been cloned during the replication process, termed clonal selection. As such, BAC clones permit an assessment to be made not only of the genomic variation that exists within a virus isolate, but also how these differences impact on viral biology. Although the concept of quasispecies is used almost exclusively to describe RNA virus populations because of the relatively high error rate of RNA polymerase [34], sequence heterogeneity has recently been identified for BAC clones of murine cytomegalovirus and Gallid herpesvirus 2 and for plaque-purified pseudorabies virus stocks [35,36,37,38]. 

Further, a BAC clone can help ameliorate the effects of *in vitro* passage of virus isolates. There are numerous examples where attenuation of the virulence of a herpesvirus can be achieved by repeated passage in susceptible cells [39,40,41]. While this is sometimes the aim of passage, for example in the development of an attenuated strain for use as a vaccine, the apparent accumulation of mutations can make it difficult to effectively study important viral properties such as virulence. An icBAC not only allows the efficient production of many copies of virus genome, it also provides the mechanism to return to the first passage without the need for *in vivo* back-passaging in the natural host and subsequent re-isolation.

## 4. BAC Construction

The primary methodology for developing icBAC is dependent on some knowledge of the viral genome sequence. This approach was first described by Messerle *et al.* (1997) who developed a transfer vector to facilitate the use of homologous recombination to transfer a BAC vector into the genome of murine cytomegalovirus. In order to increase the likelihood of successful transfer, a selection cassette was included in the vector. This approach has been widely used to generate the majority of icBAC [9,11].

Other methods of icBAC construction have also been reported, although the utility of these techniques across a broader range of viruses has yet to be demonstrated.

Smith and Enquist (1999) generated an icBAC of pseudorabies virus by first using homologous recombination in eukaryotic cells to insert a single *loxP* site into the viral genome. This construct then recombined at this site with a plasmid containing the BAC vector [9]. 

Where available, the complete genome sequence of a virus can be used to identify unique restriction endonuclease sites that can be used to increase the efficiency of homologous recombination in host cells. This approach was used by Mahony *et al.* (2002) to facilitate the construction of an icBAC for Bovine herpesvirus 1 (BoHV-1) [12].

Recently Ooi and coworkers [42] have also demonstrated BACs maintained as linear replication-competent constructs. This was achieved by inserting a bacteriophage N15 *tos* site into a circular BAC that was then resolved *in vivo* to produce hairpin telomeres that capped the ends of the linearized BAC [42]. These linear BACs have been shown to be as amenable to recombination techniques as more traditional circular BACs [43]. This technique has not yet been applied to viral icBACs. 

Due to these handling features, it is not surprising that BAC vectors have dominated the development of infectious herpesvirus clones.

The limitation of availability of genomic sequence in the construction of herpesvirus clones has potentially been overcome by two recent reports that described the insertion of BAC vectors into viral genomes using transposon-mediated methodologies [44,45]. However, so far this method has only been reported for a murine herpesvirus-68 strain and a baculovirus shuttle vector.

The generation of recombinant viruses prior to the application of icBAC was reliant on low frequency homologous recombination in virus-susceptible eukaryotic cells [3]. Typically this would require the development of a transfer or shuttle plasmid containing the transgene of interest flanked by regions of virus sequence upstream and downstream of where the transgene was to be inserted. The transfer vector was then introduced into virus-susceptible cells followed by virus infection, and the recombinant virus was plaque-purified. Various strategies were used to increase the efficiency of this process, including the addition of selectable markers or the co-transfection of infectious viral DNA and shuttle plasmid DNA [46]. Overall these were time-consuming and labour intensive methods, and due to these limitations, the focus turned to utilizing the powerful recombination machinery of bacteria.

The large size of DNA inserts in BACs limits the efficient application of traditional restriction endonuclease and ligation techniques to introduce mutations, however some methods involving restriction enzymes have been successfully utilized. RecA-assisted restriction endonuclease (RARE) cleavage involved protecting a specific restriction site by using a complementary oligonucleotide that prevented methylation when bound to the complementary sequence [47]. After dissociation of the complex, the restriction site was amenable to routine restriction and ligation procedures. This technique was used to generate a chimaeric BAC and then introduce deletions in a BAC containing the full-length LRP-1 gene [47]. *Not*I sites have been used to retrofit selectable markers into mammalian BACs due to the infrequent occurrence of *Not*I sites in mammalian genomic DNA [48]. These applications were limited by the location of pre-existing restriction sites and successful manipulation was laborious.

The application of BAC clones in the development of human disease models facilitated the development of strategies to apply bacterial genetics to increase the efficiency and precision of BAC manipulation using sequence-dependent and independent technologies. BAC engineering of viral icBACs is now common practice both to elucidate gene function and for the generation of recombinant viruses. Methods have been established for both site-specific changes, such as point mutations, insertions, deletions or gene fusions, and for random mutagenesis utilizing transposable elements. In contrast to the use of homologous recombination to generate recombinant viral progeny in eukaryotic cells, mutated genomes within icBAC can be fully characterized before attempting to recover infectious virus, avoiding time-consuming selection steps [4]. Following transfection of the modified icBAC into permissive eukaryotic cells, infectious recombinant virus may be rescued. Existing methodologies continue to be improved to enhance the efficiency of recombinant virus production and to remove marker and BAC vector sequences for use in transgenic studies.

## 5. Bacterial Genomic Recombination

Mutagenesis of BACs within *E. coli* was originally mediated by RecA, part of the DNA repair machinery of the bacterial cell [49]. In *E. coli*, double-strand breaks of the bacterial chromosome are repaired mainly through the RecBCD pathway. During this process, bacterial RecA identifies double-strand homologous sequences and strand invasion occurs, whereby RecA unwinds the dsDNA and assimilates it to its complementary sequence. The displaced single-stranded DNA is digested by exonuclease activity and DNA ligase completes the repair process (reviewed in [50,51]). Utilising constitutive bacterial recombination pathways for BAC mutagenesis is limited because the majority of laboratory strains of *E. coli* have been engineered to be recombination negative to maintain stability of DNA inserts by preventing unwanted recombination, deletions or rearrangements. Also, linear double-stranded DNA molecules are degraded due to exonuclease activity in RecBCD *E. coli* strains. Thus for BAC manipulation, RecA-mediated homologous recombination has to occur in a temporally-controlled manner. This can be achieved by either transferring the BAC construct to a recombination positive bacterial strain for manipulation, or by transiently inducing recombination functions that are encoded by either the host chromosome or on a helper plasmid. Homologous recombination techniques allow any type of mutation, point, insertion, deletion or gene fusion, to be efficiently introduced. The probability of a recombination event occurring is dependent on the length of the homology arms, and is increased by the presence of Chi sequences within the homology arms [52]. These Chi sequences signal RecBCD exonuclease to begin the process of DNA repair that facilitates recombination. 

### 5.1. Inducible Rec-A Mediated Homologous Recombinations

**Figure 1:**
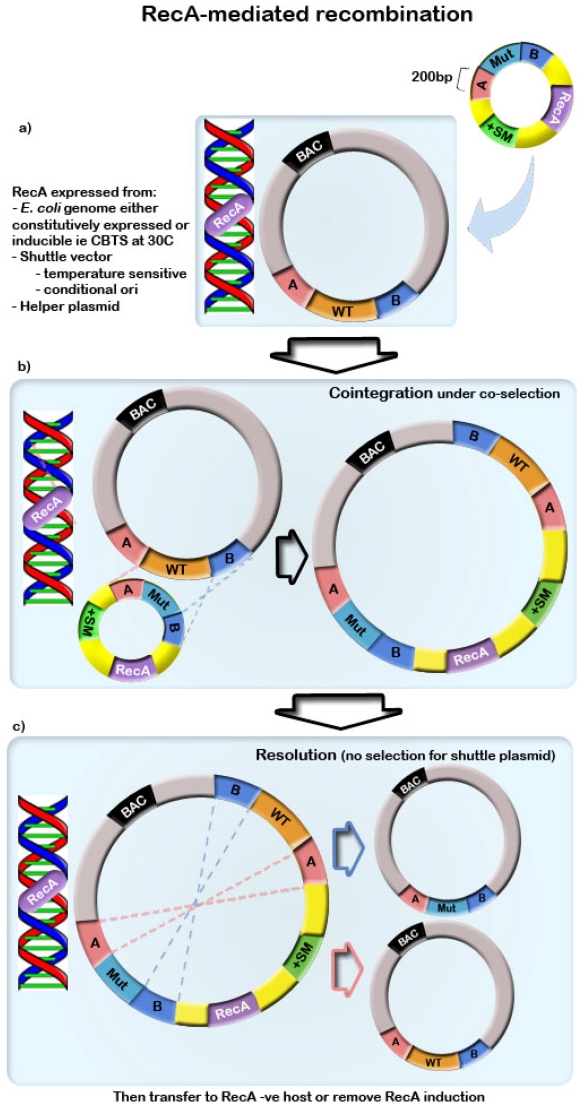
(**a**) A shuttle plasmid containing the mutation of interest flanked by homology arms >200 bp along with a positive selection marker is transformed into an *E. coli* strain containing the BAC construct. Recombination functions are induced, either from the *E. coli* chromosome or from a conditional plasmid. (**b**) Co-integration of the shuttle plasmid occurs via a single crossover event at one homology arm, and is selected for under positive selection. (**c**) Resolution occurs under selection for the BAC construct only via a second single crossover event at the remaining homology arm to result in the BAC construct containing the mutation of interest.

The RecA-mediated recombination process involves co-integration of the shuttle plasmid, usually containing positive or negative selection markers, via homologous recombination of sequences specifically targeting the transgene [2,4]. This can be a two-step ‘pop-in/pop-out’ process in which the co-integration step is followed by resolution, to remove the selection marker and other operational sequences as outlined in Figure 1. This is based on the technique originally developed for manipulation of plasmids [2]. Alternatively, to achieve gene disruption, only one step co-integration is required [53]. The mutation of interest, with homology arms no shorter than 200 bp to 500 bp, is cloned onto a circular shuttle plasmid. The mutation can be generated by restriction digestion and ligation or by PCR using primers to synthesize the homology arms, and is ligated to the shuttle plasmid using standard methods. The use of a circular plasmid rather than a linear DNA fragment is necessary to prevent degradation of linear DNA by cellular RecBCD exonuclease [54]. When recombination functions are induced, this shuttle plasmid then co-integrates into the BAC by a single crossover event with one homology arm. These co-integrates undergo dual positive selection for the markers encoded for on the BAC and on the shuttle plasmid. In a second single crossover event, resolution occurs via recombination at the second homology arm to produce either the wild-type BAC or the desired recombinant. Recombinants must be identified, usually by counterselection. Linear DNA substrates can be used for RecA-mediated recombination only in recBCD negative *E. coli* strains [55,56], however this occurs only at low efficiency [57]. 

Recombination functions can be induced in various ways. *E. coli* strains such as the CBTS strain generated by Dr O’Connor are conditionally RecA-positive [58]. In this particular strain, recombination is active at 30 °C, but is lost at temperatures higher than 37 °C [58]. Alternatively, the RecA gene can be incorporated into the shuttle vector. This method was developed by Yang *et al.* (1997) who produced pSV1.RecA [49]. The plasmid is temperature sensitive and is lost at restrictive temperatures above 42 °C. By expressing RecA from a conditional plasmid, recombination can be induced in recombination-deficient strains and the plasmid can be easily lost to minimize the recombination window and maximize stability [49]. Other conditional plasmids have also been used, including pSC101 which is temperature sensitive similar to pSV1.RecA, ColE1 plasmids which are unable to replicate in polA mutant *E. coli strains* and *pir-*dependent plasmids which have a conditional R6Kγ origin of replication [59,60,61]. RecA-mediated recombination has a number of disadvantages including the need for intensive construction of building and shuttle vectors and long homology arms [62]. Also, RecA is activated for a relatively long time period when compared directly with more recent recombination methods and this extended recombination window may compromise stability [63]. Jessen *et al.* [64] aimed to improve efficiencies of RecA-dependent recombination by incorporating precisely placed Chi sites in the shuttle vector, however Chi-stimulated recombination is generally inefficient despite long regions of homology [57]. A key disadvantage of RecA-mediated recombination is the laborious construction of the shuttle plasmid. In an effort to improve the efficiency of vector construction, Misulovin *et al.* [65] developed a method to modify shuttle vectors by PCR. Homology arms were generated from the BAC template using primers with overlapping regions. PCR products from this first step were subjected to a second round of multi-template PCR to generate a hybrid product which was then cloned into a shuttle vector. This process reduced the time required for construction of the shuttle plasmid when compared to restriction enzyme-based cloning techniques, however despite these modifications, alternative recombination systems have been developed that offer further advantages over RecA-dependent systems and are now preferred. 

### 5.2. ‘Recombineering’ Methods

To improve the efficiency and stability of BAC manipulation, strategies were developed that utilize the naturally occurring recombination functions from various bacteriophages. Murphy *et al.* [57] reported the utilization of the recombination mechanisms of the bacteriophage λ Red operon, while Zhang *et al.* [66] demonstrated the successful application of the recombination functions from the cryptic prophage Rac. The use of phage-derived recombination functions was later termed recombinogenic engineering, or recombineering, and is now widely used for BAC engineering [67].

**Figure 2:**
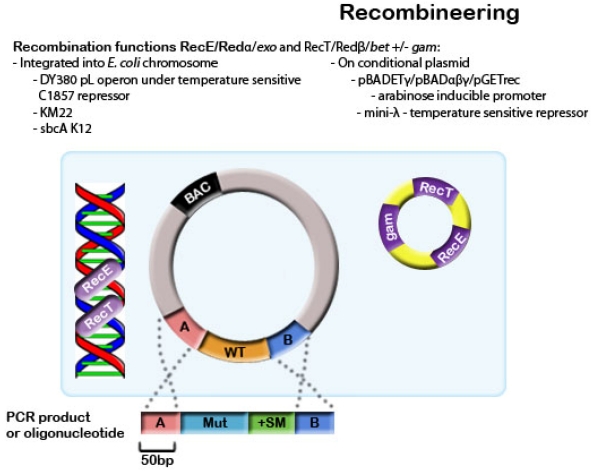
A PCR product or oligonucleotide with 50bp homology arms is transformed into an *E. coli* strain containing the BAC construct. Recombination functions are induced, either from the *E. coli* chromosome or from a conditional plasmid. The mutation is integrated by a double-crossover event, and is selected for by the positive selection marker.

Recombineering involves integrating a linear DNA molecule (transgene) into the target BAC clone at a defined location. This process is facilitated by the use of homologous sequences between the two DNA molecules, resulting in a one-step double crossover event as demonstrated in Figure 2. 

Both phage systems utilize linear DNA in the recombination process as opposed to circular DNA in the RecA-dependent system described previously [57,66]. Linear DNA can be either a double-stranded PCR product or short single-stranded oligonucleotides [57,66,68]. Efficiencies are 10 to 100 times higher when using these oligonucleotides when compared with double-stranded DNA and construction is simpler [62,69,70]. When using oligonucleotides, the lagging strand produces higher recombination efficiencies than the leading strand [68]. Oligonucleotides can also be used for recombination without selection. This has been demonstrated by Swaminathan *et al.* [71] who screened recombinants by PCR amplification of the mutated region on pooled cultures. 

#### 5.2.1. Bacteriophage λ Red Recombination

The bacteriophage λ Red recombination genes are Redα (Exo) and Redβ (Beta). The molecular mechanisms behind this form of recombination have been described elsewhere [72,73]. More recently a new model for double-stranded DNA recombination has been proposed, termed beta recombination, which may have future implications on Red recombineering [74]. Redα is a 5' to 3' exonuclease which when activated, results in the production of a single-stranded 3' overhang that is the binding site of Redβ [54]. Redβ is a single-stranded binding protein that promotes annealing of complementary strands of DNA [75]. In the Rac prophage, recombination occurs via RecE and RecT, which are homologous to Redα and Redβ respectively, and hence this system is termed ET cloning [66,76]. When recombineering using single-stranded oligonucleotides Redα function is not required, and only Redβ function is necessary [68]. The addition of Gam (γ) from bacteriophage λ to the basic phage recombination functions has led to increased efficiency of the system by protecting linear DNA from host RecBCD exonuclease activity, and it is now standard to provide *gam* with these other functions [57]. An important advantage of the phage-based recombination systems is that in contrast to the RecA system, only short DNA sequences from around 50bp in length are required to target the transgene to the site of interest, and these can be simply constructed using PCR methods [66]. Recombineering is simple, precise, rapid, efficient and inexpensive and has now become the cornerstone for recombinant BAC generation [77]. One drawback of this approach, however, is that the genomic sequence of the target region must be known; therefore its application in manipulation of uncharacterized viral genomes can be limited.

Murphy *et al.* [57] was the first to demonstrate λ phage recombineering. Red recombination functions were initially expressed from a multi-copy plasmid but were subsequently integrated into the *E. coli* KM22 chromosome through replacement of chromosomal RecBCD with the P*_lac_-Red* operon. This system was RecA-independent, in that recombination could occur in the complete absence of RecA, however efficiencies and cell survival have been demonstrated to be improved when RecA is expressed concurrently [45]. Efficiencies from the plasmid-based expression system were 15–130 times higher than those seen in recombination proficient *E. coli* strains, and were higher again when recombination functions were expressed from the bacterial chromosome [57]. Because of the improved efficiencies, Red recombination has also been used to simultaneously insert two selectable cassettes concurrently at individual loci [78]. No cloning of vectors is required and the method is mobile and applicable to other *E. coli* strains via P1 transduction. Similarly, Yu and coworkers [69] constructed *E. coli* strains containing a defective λ prophage integrated into the bacterial chromosome, however the intact *p*_L_ operon in these strains was under control of the temperature-sensitive λ cI857 repressor. At 32 °C, the repressor is active and recombination functions are inhibited, while at temperatures above 42 °C, the repressor is inactivated and recombination can proceed [69]. Due to the strength of this promoter, 15 minutes was ample time for recombination to occur, and approximately 1 in 500 clones carried the desired mutation [69]. Because recombination functions are expressed under the control of the natural promoter, this system is very efficient, tightly regulated and genes are expressed in their physiological molar ratio [79]. This temperature-sensitive repressor system was also constructed on a non-replicating circular plasmid termed mini-λ [80]. A range of other conditional plasmids, both high and low copy number, carrying recombination functions have also been used, similar to those described above for RecA-dependent recombination [79,81,82]. The use of plasmid constructs to provide recombination functions limits the range of plasmids that can be used for subsequent manipulations [69]. However an advantage of the plasmid-based system is the ease of mobility between strains, while some strains in which the recombination functions have been integrated into the host chromosome can be difficult to transform [83].

#### 5.2.2. ET Cloning

ET cloning was first demonstrated by Zhang *et al.* (1998) in sbcA K12 *E. coli* hosts that contain the Rac prophage integrated into the *E. coli* chromosome and therefore, express RecE and RecT recombination functions [66]. In these hosts, the sbcA mutation activated the RecET recombination pathway. To enable this system to be mobile, the plasmid pBAD-ETγ was then created [66]. In the pBAD system, RecE is under control of the arabinose-inducible promoter pBAD, while RecT is under control of the strong constitutive promoter EM7 and *gam* is under the constitutive Tn5 promoter. Replacement of RecE and RecT with λ Redα and Redβ gave rise to pBAD-αβγ, which was shown to be one to three times more efficient than pBADET-γ [76]. The constitutive expression of *gam* has since been shown to reduce cell viability and may limit recombination efficiency [84,85]. Because of this, an ET cloning vector was modified to create pGETrec [86]. In this plasmid, recE, recT and *gam* expression are all under the control of the pBAD promoter and hence expression of all three genes is tightly regulated. Published reports state that the plasmid is rapidly lost from bacteria once positive selection for the plasmid is removed [10,76]. ET recombination has also been used for sub-cloning from BACs to plasmids at high efficiency, although some background from re-circularisation of ‘empty’ plasmids has been reported [87]. Bacteriophage P22 recombination functions encoded by *arf*, *erf*, *abc1* and *abc2* have also been demonstrated to stimulate recombination, however efficiencies were only 5 to 10% that of λ phage Redα and Redβ [57,88]. 

### 5.3. Counterselection Methods

**Figure 3:**
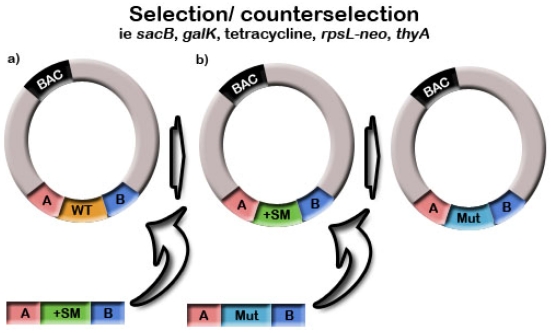
(**a**) A positive selection marker is integrated into the BAC construct using homologous recombination and recombinants are isolated under positive selection. (**b**) A second round of homologous recombination is used to replace the positive selection marker with the mutation of interest. Recombinants are selected for under negative (counter-) selection.

To identify recombinant clones, selection and counterselection methods, also referred to as positive/negative selection, are frequently used. Figure 3 outlines an insertion into a BAC using selection and counterselection. This process allows scarless removal of operational sequences such as selection markers, as well as aiding in identification of recombinant clones. Various methods have been used, however counterselection is generally much less efficient than positive selection due to a large number of false positive background colonies. This occurs because any mutation in the counterselection gene that reduces gene expression will be selected for [66]. 

Common counterselection methods used for BAC mutagenesis include fusaric acid, *neo-sacB*, ccdB, *rpsL*-*neo* and *galK* [49,63,66,89]. Original recA-dependent recombination frequently used tetracycline resistance as a positive selection marker, followed with counterselection by bacterial growth on plates containing fusaric acid [49]. Colonies that retained the tetracycline resistance marker were unable to grow on fusaric acid medium, while those that had lost the tetracycline marker did grow [90]. However, growth was slow and this selection procedure showed only 4% efficiency during resolution [66,91]. *S*ucrose-based counterselection against *sacB* has been widely reported, however it has been noted that spontaneous mutations occurred frequently (~1 in 10^4^) in the *sacB* region, resulting in selection for these mutations and thus significant background which requires further characterization to identify true recombinants [77,92,93]. For example, Muyrers *et al.* [92] reported that up to 90% of colonies after counterselection were false positive colonies. An additional drawback of this system is the poor efficiencies for transfer vector construction due to the large size (3 Kb) of the *sacB* gene [77]. *RpsL*-based counterselection for BAC mutagenesis was initially demonstrated by Imam and coworkers [63]. Specific *rpsL* mutant strains, when provided with the wild-type *rpsL* allele, become streptomycin-sensitive but resistant to a second antibiotic through a second selectable marker [94,95]. Resolved recombinants then returned to the streptomycin-resistant state when the *rpsL* allele was replaced. This selection method is only applicable to specific streptomycin-resistant *rpsL* bacterial strains, although the common BAC host strain DH10B strain is suitable. Spontaneous mutants are rare compared to *sacB* counterselection and selection is very efficient [63,96,97]. Counterselection has also been achieved by using a cassette containing a I-*Sce*I restriction site and antibiotic resistance marker [98]. The cassette was replaced by a PCR product with the desired mutation, and after digestion with I-SceI, recombinants were selected as those that did not linearise. This technique was also prone to spontaneous mutations in the restriction site, resulting in a high background of false positive clones [89,98].

Another approach used *E. coli* strains that have had the galactose operon deleted to allow *galK* gene-based selection [89]. *galK* was first inserted into the BAC construct and recombinants were selected for by growth on media containing galactose as the sole carbon source. *galK* encodes the galactokinase enzyme which is necessary for galactose utilisation [89]. Galactokinase also phosphorylates 2-deoxy-galactose (DOG) which upon further metabolism, produces the toxic end product 2-deoxy-galactose-1-phosphate [89]. The *galK* gene was replaced by the foreign DNA insert via homologous recombination. Loss of the *galK* gene, hence insertion of the foreign DNA, was then screened for by growth on media containing DOG and glycerol. Non-recombinants still containing the *galK* gene were selected against by the formation of toxic 2-deoxy-galactose-1-phosphate. *galK* selection is useful because it can be used for both positive and negative selection, there is minimal background, the *galK* cassette can be recycled and the cassette is small, which simplifies development of transfer constructs [89]. However counterselection against *galK* is inefficient, it is only suitable for use in specific *galK-*negative *E. coli* strains and PCR screening of potential recombinants is required because of the risk of spontaneous mutations [77,99]. The use of thymidylate synthase A (*thyA*) shares similar advantages and disadvantages with *galK* selection [100]. Specific *thyA*-null *E. coli* strains, such as QW1, are required and the *thyA* gene is first integrated and then replaced to obtain the desired recombinant [100]. In the presence of *thyA*, colonies are able to grow in a minimal medium that lacks thymine. Once *thyA* has been eliminated, recombinants are selected for in media containing thymine and trimethoprim.

## 6. Site-Specific Recombinases (SSRs)

**Figure 4:**
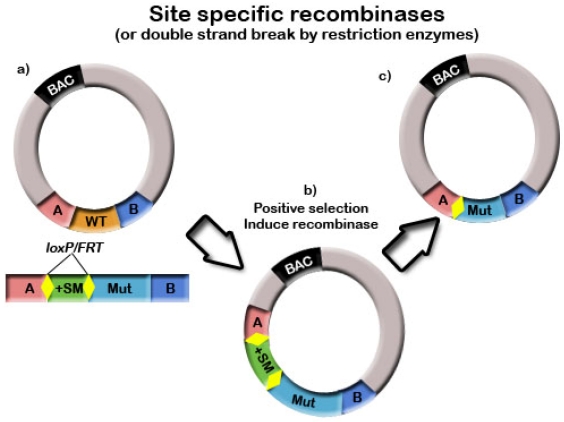
(**a**) The mutation to be inserted contains a positive selection marker flanked by *loxP* or *FRT* sites. This mutation is inserted into the BAC using a homologous recombination method. (**b**) Once the mutation has been integrated into the BAC and selected for using the positive selection marker, the site-specific recombinase is induced. (**c**) This results in removal of the positive selection marker, leaving a single persistent *loxP* or *FRT* site.

Site-specific recombinases (SSRs) have been used in conjunction with homologous recombination techniques in bacteria to further manipulate and produce recombinant BACs. SSRs have been used to delete sequences such as prokaryotic regulatory sequences, selection markers and the BAC vector backbone. This is discussed in further detail in Section 7Section 7. SSRs have also been used to insert sequences into BACs (see Figure 4) [101]. Deletions occur at higher efficiencies than insertions, as insertions are kinetically unfavourable, however SSRs are still very useful tools and large insertions have been achieved [102]. The most common SSRs used are Cre recombinase and Flp/Flpe recombinase. These recombinases target specific *loxP* or *FRT* sites respectively, and efficiencies are extremely high [67,76,103]. By flanking sequences to be removed, such as BAC vector sequences, with recognition sites in the same orientation, upon expression of recombinase functions, intervening sequences are looped out and excised [9,26,78,104,105]. 

Insertions can be created by co-integrating a shuttle plasmid such as pRETRObac into the BAC clone via a single *loxP* site, however this can only occur at one location on the BAC vector and thus is not useful for specific gene modifications [101]. The ‘flip-flop’ and HSVQuik systems for herpes simplex virus have also used site-specific recombination for the creation of BAC vectors and insertion mutagenesis [106,107]. The flip-flop system developed by Kuroda *et al.* utilized both Cre and Flpe recombinase to first integrate the BAC vector and mutation of interest into HSV and then to remove the BAC vector backbone [106]. It was specifically designed so that non-recombinants exceeded the maximum packaging capacity of the virus and were therefore unable to grow, simplifying the screening process [106]. The HSVQuik system from Terada *et al.* [107] similarly used sequential site-specific recombination to first insert the transgene into the BAC in bacteria and then to remove the BAC backbone in eukaryotic cells [107]. In this system, typically 90% of resultant virions correctly had the BAC backbone removed [107]. 

## 7. BAC De-Engineering

A major potential concern of BAC engineering, particularly for *in vivo* transgenic or vaccine applications, is the presence of residual BAC and marker sequences in the final recombinant. Various methods have been developed for markerless BAC mutagenesis, including the use of site-specific recombinases as detailed above [4,9,15,17,19,20,22,26,32,66,83,104,108,109,110]. One of the disadvantages of using SSRs, particularly in transgenic studies, is the persistence of a single 34 bp restriction site or ‘scar’ that remains after recombination has occurred [108]. However the presence of this small scar in viral vectors or engineered vaccines is generally not a concern. Wagner *et al.* [111] and Tischer and coworkers [112] utilized sequence duplications on the insertion DNA to delete BAC sequences by stimulating recombination once reconstituted in eukaryotic cells. Markerless constructs have also been created through co-targeting of a selectable marker to the *E. coli* chromosome [113]. This was achieved by using an excess of the BAC targeting cassette, which resulted in a high probability that those colonies containing the selectable marker in their chromosome would also contain a mutation in the BAC they harbor. Pofsai *et al.* [114] co-integrated the mutation of interest into the host chromosome and subsequently utilized the homing endonuclease I-SceI to create a double-strand break at a pre-engineered restriction site. This stimulated intramolecular recombination, and recombinants were screened for by allele-specific PCR, eliminating the need for selection markers. Recombination efficiencies varied, but were low for large deletions, and high efficiencies were only achieved with repetitive colony selection [114]. Herring and coworkers [115] used a similar technique they termed “gene gorging”. This required a donor plasmid carrying the mutation of interest to be linearised with I-SceI *in vivo* to stimulate Red recombination functions. These were provided on separate mutagenesis plasmids. Again no selection was required and screening for recombinants was by PCR [115]. Tischer *et al.* [116] refined this method, later termed “en passant” recombineering, by using I-SceI cleavage to provide the substrate for additional recombination steps. Subsequent intramolecular recombination of sequence duplications resulted in removal of the selectable marker. Assisted large fragment insertion by Red/ET-recombination (ALFIRE) also utilized I-SceI [117]. Other homing endonucleases such as I-CeuI have been used for BAC mutagenesis, for example in the Homingbac baculovirus cloning system [118]. Parent Homingbac baculoviruses were pre-engineered to contain a eukaryotic *GFP* fluorescent marker gene. Digestion with I-CeuI resulted in excision of this *GFP* marker and transgene DNA with compatible end sequences could then be ligated into the parent baculovirus [118]. Although unlikely to be 100% efficient, background parental virus could not be detected in this study. 

## 8. Random Mutagenesis

While site-specific mutagenesis techniques are necessary to target specific genetic loci, these techniques are not suited for global genome analysis. In contrast, random mutagenesis is particularly useful for the rapid generation of libraries of gene disruptions. These libraries can then be screened to identify mutations in specific genes and to elucidate the effect of the mutation on virus function. The most widely used random mutagenesis methods rely on transposon technologies to achieve gene disruption. Transposons are mobile genetic elements originally discovered in maize by McClintock [119] and multiple transposon families have since been characterized . These elements can be activated by a transposase to induce movement and can be engineered to contain SSR recognition sites, foreign DNA, primer binding sites, regulatory elements and antibiotic selection markers between their end sequences [120]. Although many transposon families exist, the most useful and efficient for BAC modification are those that preferentially insert into negatively supercoiled plasmid DNA [121,122]. The major advantages of transposon mutagenesis are that the target sequence does not have to be known, only very small amounts of DNA are required and a large number of mutants can be rapidly generated, which can then be screened to identify the insertion site of the transposon [123]. A disadvantage of this method is that multiple insertions can occur leading to deletions and rearrangements of the BAC construct, however this can be controlled by varying the insert to vector ratio [123]. Because of the advantages of this technology, transposable elements occupy a unique niche in BAC mutagenesis.

Although transposons were used extensively preceding the development of BACs, including for mutagenesis of herpes simplex virus 1 [124], the technology was first applied to BAC constructs in 1999. Brune and coworkers [125] utilized Tn1721, a member of the Tn3 transposon family, in a mini-transposon system called Tn*Max*, originally developed in 1993 [121], to rapidly identify essential and non-essential genes of murine cytomegalovirus. The transposon construct was delivered into *E. coli* harbouring the BAC construct on a temperature-sensitive plasmid, and at permissive temperatures transposition occurred [125]. Simultaneously, Smith *et al.* [9] used a mini-Tn5 transposon on a delivery plasmid for mutagenesis of a pseudorabies virus BAC clone and this technology was later also applied to a human cytomegalovirus BAC clone [126]. These systems relied on transposition occurring within bacteria, and required further characterization to ensure the transposon had inserted into the BAC and not into the bacterial chromosome. In 2004, an *in vitro* Tn5 transposition system was used to create a gene disruption library of a bovine herpesvirus-1 BAC clone [12]. This utilized a hyperactive Tn5 system developed for mutagenesis of plasmids by Goryshin in 1998 [127]. Because the reaction is performed *in vitro*, transposition only occurs into target BAC DNA and mutants can then be easily isolated after transformation into bacteria. Other transposition systems used for random mutagenesis of herpesvirus BACs have included a Tn3-based system for mutagenesis of a murine cytomegalovirus BAC and the use of MuA transposition for a bovine herpesvirus-4 BAC [128,129]. Transposition methods have been used to produce nested deletions and small insertions, however the major use of these transposon systems is the rapid creation of gene disruption libraries [130]. 

Transposon technology can also be applied to targeted BAC mutagenesis. The Tn7 transposon is site-specific, targeting *attTn7* sites when transposition is activated [131]. In initial studies when applied to cytomegalovirus BACs, partial and complete deletions were reported to occur frequently [132]. However, others have used Tn7 transposition systems with great success [3,133,134]. Recently, Tn7 has again been used for site-specific transposition by introducing a Tn7 transposon target site into a varicella vaccine virus BAC by RecA-mediated recombination and subsequently introducing foreign sequences by site-directed transposition [135]. Currently, transposition-based technologies are the system of choice for global mutagenesis studies of herpesviral genes due to the rapid speed in which a large number of mutants can be generated. 

## 9. Conclusions

The development of BACs has revolutionized molecular cloning of large DNA molecules and they are now widely used in many applications, including investigations into herpesvirus biology. The stability, large insert size and ease of manipulation of the BAC constructs within the *E. coli* host has rapidly led to the preference for BACs over YACs and cosmids. Protocols for cloning viral genomes as BACs are now widely published. The use of homologous recombination, using both λ Red recombination and ET cloning, allows precise site-specific mutagenesis for characterization of viral genes and genetic engineering of recombinant virus strains. This has widespread implications in delivery of therapeutics, particularly in the area of DNA vaccine development. Alternatively, BACs can support rapid characterization of viral genes through random transposon mutagenesis. This is particularly useful in initial studies into newly isolated, unstudied viruses where genomic sequence data is still unknown. BACs are currently widely used in transgenic and knock out studies and are being used to monitor gene expression and target specific tissues *in vivo*. Due to the diverse applications for BACs, they will continue to be a cornerstone of virological research. 
